# The Multifaceted Role of Th17 Lymphocytes and Their Associated Cytokines in Cancer

**DOI:** 10.1155/2013/957878

**Published:** 2013-12-28

**Authors:** Darya Alizadeh, Emmanuel Katsanis, Nicolas Larmonier

**Affiliations:** ^1^Cancer Biology Graduate Interdisciplinary Program and Department of Pediatrics, Steele Children's Research Center, University of Arizona, 1501 N. Campbell Avenue, P.O. Box 245073, Tucson, AZ 85724-5073, USA; ^2^Department of Immunobiology, BIO5 Institute and Arizona Cancer Center, University of Arizona, Tucson, AZ 85724, USA

## Abstract

While the role of T helper 17 lymphocytes (Th17) in the pathogenesis of autoimmune diseases and in infectious immunity has been relatively well defined, the impact of these cells and their associated cytokines on cancer development is still under debate. Although multiple reports have indicated that Th17 can promote anticancer immunity, others have argued that these cells may exhibit tumor-promoting properties. This dichotomy in the function of Th17 lymphocytes in cancer may be related to the versatile nature of these cells, being capable of differentiating into either proinflammatory Th1 or suppressive FoxP3-expressing Treg cells or hybrid T cell subsets depending on the underlying environmental conditions. In the current review, we examine the role of Th17 lymphocytes and Th17-associated cytokines in cancer and discuss how factors that control their final lineage commitment decision may influence the balance between their tumor-promoting versus tumor-suppressing properties.

## 1. Introduction

CD4^+^ T helper (Th) lymphocytes are essential for the regulation of immune responses as they are endowed with the ability to modulate the function of CD8^+^ cytotoxic T lymphocytes (CTLs) [1, 2], B cells [3], NK cells [4], macrophages, and dendritic cells [5, 6]. Following triggering of their T cell receptor (TCR) and in the presence of appropriate costimulatory signals and specific cytokines, naïve CD4^+^ T lymphocytes differentiate into various effector or regulatory cells characterized by distinct functions and specific cytokine production profiles. For many years, it was believed that the expression of two mutually exclusive differentiation programs led to the polarization of naïve CD4^+^ T cells towards either Th1 or Th2 lymphocytes [7, 8]. Terminally differentiated Th1 cells are characterized by the expression of the transcription factor Tbet and the production of IFN*γ* [9]. Th1 activate CTLs, macrophages and are required for the elimination of intracellular pathogens [7, 10]. Th1 cell lineage commitment is primarily triggered by IFN*γ* and IL-12 [11, 12]. Th2 lymphocytes, defined by transcription factor GATA3 expression and the secretion of IL-4, IL-5, IL-10, and IL-13, play an essential role in B cell-mediated humoral responses against extracellular pathogens and can inhibit Th1-dependent cellular immunity [13–15]. More recently, several subsets of CD4^+^ T cells exhibiting immunosuppressive activity have been described (extensively reviewed elsewhere [16–21]). These so-called regulatory T lymphocytes (Tregs) may be generated during T cell development in the thymus (naturally occurring Treg) or may be induced in the periphery from naïve CD4^+^ T cells (induced/adaptive iTreg) [22–26]. Treg generation essentially depends on transforming growth factor *β* (TGF*β*), together with TCR, costimulatory signals, and IL-2 [27–29]. Extensive studies have demonstrated that the forkhead/winged helix transcription factor FoxP3 is fundamental for the development and function of Treg and remains one of the most specific molecular markers for these cells [21, 24]. Treg efficiently suppress effector T lymphocytes and may inhibit the function of B, NK, dendritic cells, or macrophages through different mechanisms [22]. They are, therefore, essential components of the regulatory networks controlling autoimmunity, infection, or cancer [30, 31].

In recent years, T helper 17 lymphocytes (Th17) have emerged as a new distinct effector CD4^+^ T helper cell subset, prompting revision of the Th1/Th2 paradigm. Th17 produce large quantities of IL-17 and exhibit effector functions distinct from Th1 and Th2 lymphocytes. They play an important role in the clearance of pathogens that are not adequately handled by Th1 or Th2 lymphocytes. Th17 cells are potent inducers of tissue inflammation and have been identified as major contributors to the pathogenesis of multiple autoimmune conditions in animals and humans [32–34]. However, the role of Th17 in cancer is still being intensively discussed, with conflicting reports related to the pro-versus antitumoral effects of these cells. This discordance may be explained by different cytokine signature profiles inherent in the high degree of plasticity of these cells. We provide an overview on the requirements for Th17 development and the direct or indirect impact of Th17 lymphocytes and the cytokines they produce on antitumor responses.

## 2. T Helper 17 Lymphocytes: Cytokine Signature and Differentiation Profile(s)

### 2.1. Th17 Differentiation

Th17 cells are defined as CD4^+^ T lymphocytes secreting substantial amounts of interleukin 17A (IL-17A) and expressing the transcription factor retinoic acid receptor-related orphan receptor gamma t (ROR*γ*t), which seems to act as a molecular determinant for their polarization [35, 36]. In addition, Th17 produce IL-21 and IL-22 [37] and, depending on the differentiation/environmental conditions secrete variable amounts of TNF*α*, IFN*γ*, and/or GM-CSF [32, 38–41]. Th17 foster B lymphocyte-mediated immunity [3], contribute to the migration and activation of macrophages [42], neutrophils [43], and regulate the activation and expansion of CD8^+^ T cells [41, 44].

**Figure 1:**
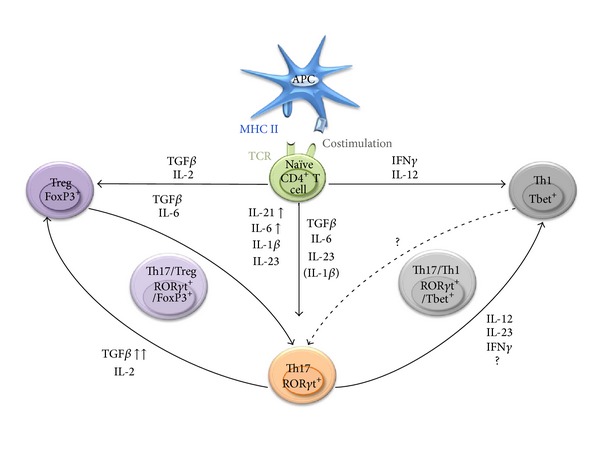
Specific cytokines drive the differentiation of specialized T helper lymphocytes. Naïve CD4^+^ T lymphocytes, upon activation and in the presence of specific cytokines, differentiate into Th1, Th2, Th17, or Treg. The plasticity of Th17 and Treg enables them to transdifferentiate into Th17/Treg subsets. Th17 cells can also acquire a Th1-type phenotype leading to “hybrid” Th17/Th1 cells. The nature and concentration of the cytokines present in the differentiation milieu lead to the activation of distinct signaling cascades and transcription factors which control the developmental program of these specific lineages.

Th17 can be generated *in vitro* from naïve CD4^+^ T lymphocytes using specific cytokines. In mice, the combination of TGF*β*, IL-6, and IL-23, in presence of TCR and CD28 signals (antigen presenting cells, plate-bound anti-CD3 plus anti-CD28 or anti-CD3/anti-CD28 coated microbeads), is required and sufficient to drive the differentiation of naïve CD4^+^CD25^−^ T cells into Th17 (Figure 1) [45]. Studies have indicated that IL-6, by inhibiting TGF*β*-driven induction of FoxP3, impairs Treg differentiation, leading to IL-17-producing ROR*γ*t^+^ lymphocyte generation. However, other reports have also shown that, in IL-6^−/−^ mice, Treg depletion increases the susceptibility of the animals to experimental autoimmune encephalomyelitis (EAE) as a result of enhanced pathogenic Th17 responses [39, 46]. These last observations suggest that Th17 lymphocytes can be generated in absence of IL-6. IL-21 was further identified as an alternative proinflammatory cytokine capable of suppressing TGF*β*-mediated induction of FoxP3 expression in the absence of IL-6 [39, 47]. IL-21, produced in large amounts by Th17, promotes an autocrine amplification feedback loop enhancing Th17 generation especially in the absence of IL-6 [48]. The IL-23 receptor (IL-23R) is composed of IL-23R and IL-12R*β*1 [49]. Naïve CD4^+^ T lymphocytes express very low levels of IL-23R. Conversely, Th17 are characterized by the expression of the IL-23R. It is therefore not surprising that, although IL-23 is not required for the initial Th17 lineage commitment, this cytokine fosters Th17 expansion and survival and contributes to their stabilization and proinflammatory properties [50]. Indeed, in IL-23p19-deficient mice, the number of Th17 is substantially decreased compared to their wild-type counterparts [51, 52]. In addition, IL-23 appears essential for the pathogenic properties of Th17 as demonstrated in collagen-induced arthritis (CIA) and EAE models [51, 53]. IL-23 is also essential for the generation of Th17 in prolonged *in vitro* cultures [50]. The proinflammatory cytokine IL-1*β* has also been reported as another important factor in the polarization of Th17 cells in proinflammatory environments. IL-1*β* induces interferon regulatory factor 4 (IRF4), which is a critical regulator of the IL-21 autocrine signaling loop [54, 55].

In humans, the conditions that would drive optimal Th17 differentiation remain unclear. Several reports have indicated that TGF*β* may not be necessary for the generation of these cells [56–58] while other studies have argued for a critical role of this cytokine in Th17 differentiation [47, 59, 60]. A study by Yang et al. indicated that the combination of TGF*β* with IL-21 but not IL-6 was effective in inducing Th17 differentiation [47]. Other reports have suggested that IL-1*β* alone or in combination with TGF*β* is also required for human Th17 production [61]. Similar to the observations made in mice, the addition of IL-23 supports Th17 proliferation and stabilization [62].

### 2.2. Th17 Plasticity

Th1 and Th2 cells are relatively stable and terminally differentiated subsets: they essentially do not transdifferentiate into other specialized CD4^+^ T helper cell lineages. On the other hand, one of the most striking characteristics of Th17 is their high degree of plasticity and their remarkable ability to give rise to other populations of either proinflammatory effector cells such as Th1 [63] or immunosuppressive FoxP3^+^ Treg [64]. Interestingly, Th17 may themselves originate from FoxP3^+^ Treg cells that have undergone “reprogramming” in specific environmental conditions [65]. Intermediary cell subpopulations expressing both FoxP3 and ROR*γ*t and demonstrating immunosuppressive activity have been identified [66].

TGF*β* appears as a master regulator of the balance between Th17 and suppressive Treg differentiation. The role of TGF*β* in Th17 polarization has, however, been questioned. Although some studies have indicated that TGF*β* is required for the production of IL-17 by Th17 cells [60], others have reported that TGF*β* may not be essential for the induction of Th17 [58]. Additional reports have demonstrated that the generation of Th17 and the development of Th17-mediated EAE are impaired in transgenic animals with T lymphocytes deficient in functional TGF*β* receptor [67] or when TGF*β* expression is ablated in T cells [68]. TGF*β* alone induces the expression of FoxP3 and ROR*γ*t [69]. However, in the presence of IL-6, IL-21, or IL-23, FoxP3 expression is inhibited while ROR*γ*t expression is induced, resulting in Th17 generation instead of Treg. While it has been established that TGF*β* is required for the initial production of IL-17 and for the induction of IL-23R expression [47, 57, 60], high concentration of TGF*β* conversely impairs the expression of IL-23R [69]. Therefore, the outcome of the balance between Treg versus Th17 generation is likely dictated by the strength of the signals provided by TGF*β* and thus depends, at least partially, on the concentration of this cytokine in the environment of the differentiating cells. Large amounts of TGF*β* primarily promote the development of cells endowed with immunosuppressive activity (possibly even in the presence of low doses of IL-6 or IL-21), while intermediary or low concentration of TGF*β* in combination with the proinflammatory cytokines IL-6 or IL-21 drives primarily the differentiation of naïve CD4^+^ T cells into Th17 (Figure 1). This scenario may explain the observed Th17-Treg plasticity. Recent reports have also indicated that, in specific conditions, fully differentiated FoxP3^+^ Treg may undergo “reprogramming” into effector helper T cells. These reprogrammed Treg are not immunosuppressive, produce proinflammatory cytokines (among which IL-2, TNF*α*, or IL-17), and may play an important role in anti-tumoral CD8^+^ T cell activation [70]. Importantly, it has been shown that reprogrammed Treg may lose or maintain FoxP3 expression [70, 71]. Additional studies have reported that, in presence of IL-2 and IL-1*β*, human Th17 may be preferentially differentiated from naïve FoxP3^+^CD25^−^CD4^+^ Treg rather than from naïve FoxP3^−^CD25^−^CD4^+^ T cells [72, 73]. In these studies, Th17 differentiation was enhanced by IL-23 and TGF*β* [73].

Th17 may also redifferentiate into Th1 lymphocytes. Indeed, IL-17-producing CD4^+^ T lymphocytes expressing the Th1 lineage-specific transcription factor Tbet and producing IFN*γ* have been described (Figure 1) [74]. Tbet^+^ Th17 cells have been identified in patients with multiple sclerosis [38] and IFN*γ*-producing human Th17 cells have been described [57]. These intermediary Th17/Th1 lymphocytes have been reported as pathogenic [32, 75] and as outlined in Section 3 can exhibit anti-tumoral activity [41, 74]. Initially, the recognition that IL-23 and IL-12 shared the common IL-12p40 (IL-12*β*) subunit and the observation that IL-23 induced not only IL-17 but low amounts of IFN*γ* led to the speculation that Th17 cells developed as a distal branch of the Th1 lineage. However, a recent study reported that Th17 precursors may produce IFN*γ*, independently of IL-23 and IL-12 signaling. These Th17 precursors are also capable of responding to IL-23 and IL-12 and, in the absence or in presence of low concentrations of TGF*β*, can differentiate into cells characterized by enhanced production of IFN*γ* and minimal IL-17A and IL-17F secretion [76]. These studies confirmed that Th17 lymphocytes belong to a distinct cell lineage susceptible, however, to reprogramming into Th1 cells. This observed stability or plasticity of Th subsets seems controlled by epigenetic modifications regulating the expression of key transcription factors and cytokines specific for a dedicated Th lymphocyte lineage.

Thus, Th17 lymphocytes represent a highly heterogeneous cell population with a remarkable flexibility in their ability to differentiate into immunosuppressive Treg or effector proinflammatory Th1 depending on the environmental conditions. Since Treg are known to suppress anti-tumor immune responses and promote cancer development while Th1 enhance anti-tumoral immunity, it is therefore not surprising that Th17 have been reported to exhibit both pro- and anti-tumor activities.

## 3. IL-17-Producing Cells, Th17-Associated Cytokines, and Th17 Lymphocytes in Cancer

### 3.1. IL-17-Producing Cells, IL-17, and Major Th17-Associated Cytokines

#### 3.1.1. IL-17-Producing Cells

IL-17A belongs to the IL-17 family, composed of 6 members (IL-17A-F) [77]. Although IL-17A and IL-17F are the signature cytokines defining CD4^+^ Th17 cells, it should be noted that IL-17 is also produced by *γδ*T cells [78], natural killer (NK) T cells [79], CD8 T cells [80], macrophages [81], neutrophils, and eosinophils [82]. The role of IL-17 expressing non-CD4^+^ T cells in cancer has been examined in several studies [81, 83, 84]. For instance, the adoptive transfer of *in vitro* generated CD8^+^ T cells producing IL-17 (Tc17) in mice promoted anti-tumor immunity against B16 melanoma by fostering the recruitment of other inflammatory anti-tumoral cells such as CTL, Th1, neutrophils, or macrophages [85, 86]. In another report, mast cells accumulating in a murine hepatocellular carcinoma fostered the recruitment of myeloid-derived suppressor cells (MDSC) and induced IL-17 production by these MDSC. In turn, IL-17 secreted by MDSC attracted Treg to the tumor site and enhanced their suppressive function, therefore promoting tumor growth [84]. Additionally, tumor-associated macrophages expressing IL-17 were detected in human breast cancer tissues and their presence was directly associated with the degree of invasiveness of the tumor [81]. Whether these IL-17-producing non-Th17 cells may mediate pro- versus anti-tumoral effects does not solely depend on IL-17 as they produce variable amounts of a plethora of other cytokines with different activities. The abovementioned reports, underline the importance of distinguishing Th17 from IL-17-producing cells in general, as IL-17 targeting does not solely affect the role and function of Th17.

#### 3.1.2. IL-17

IL-17A and IL-17F have been involved in proinflammatory cytokine and chemokine release by neutrophils, leading to tissue inflammation [82, 87]. The specific role of this cytokine in the development of malignancies remains elusive. Multiple reports have provided evidence that IL-17 promotes angiogenesis [88–90] and tumor development [89, 91, 92]. However, results from IL-17 deletion or ectopic expression remain conflicting. Several studies using IL-17^−/−^ mice have demonstrated that the absence of IL-17 may promote tumor progression in mouse B16 melanoma [41] and MC38 colon carcinoma models [93]. The growth and propensity to give rise to lung metastases of MC38 tumors is augmented in IL-17-deficient mice, which is associated with decreased IFN*γ* ^+^ NK and IFN*γ* ^+^ tumor-specific T cells in the tumor draining lymph nodes and at the tumor sites [93]. IL-17^−/−^ mice bearing B16 melanoma also exhibit increased lung metastases associated with reduced numbers of CD4^+^, CD8^+^ T cells, granulocytes, and CD11c^+^CD11b^+^ and CD11c^+^CD8a^+^ DCs at the tumor sites. Additionally, the activation status of CD4^+^ T lymphocytes isolated from lung metastases was reduced [41]. Conversely, other studies performed with both B16 melanoma and MB49 bladder cancer models have argued that IL-17 deficiency resulted in reduced tumor burden [92]. A recent study has demonstrated that the growth of various tumors (EL4 lymphoma, Tramp-C2 prostate cancer, and B16-F10 melanoma) is significantly impaired in IL-17R^−/−^ mice compared to their wild-type counterparts. In this study, IL-17R deficiency resulted in an increase in intratumoral CD8^+^ T cells and reduced MDSC numbers in the tumor microenvironment. Interestingly, systemic pretreatment of animals with murine IL-17A exacerbated tumor growth [91].

Several human studies have highlighted the correlation between the level of IL-17 and poor prognosis in cancer patients [94, 95]. Increased numbers of IL-17-producing cells directly correlated to microvessel density in tumors and overall poor survival in hepatocellular carcinoma patients [94], as well as in non-small-cell lung cancer patients [95]. Consistent with these results, another study showed an increase in the level of IL-17 (most of which being secreted by CD4^+^ T cells) in melanoma, breast, and colon cancer patients. Further characterization revealed that these tumor-derived IL-17 expressing cells were not immunosuppressive, but promoted tumor growth in an *in vitro* culture system [96]. Additionally, in colorectal carcinoma patients, a significantly higher frequency of IL-17-producing CD4^+^ and CD68^+^ cells were detected within the tumors when compared to the normal tissues. High expression of IL-17 was associated with increased microvessel density [88].

The angiogenic property of IL-17 has been an additional subject of debate. Indeed, several studies have linked IL-17 production to the induction of proangiogenic factors [88, 89, 92]. An early study conducted by Numasaki et al. demonstrated that the retroviral transduction of the IL-17 gene in cancer cells (MCA205 fibrosarcoma and MC38 colon adenocarcinoma) resulted in enhanced tumor growth *in vivo* while it had no effect on tumor cell proliferation *in vitro.* Tumors transduced with IL-17 exhibited significantly higher vascular density when compared to controls. IL-17 also enhanced the formation of vascular endothelial cells. Together these results indicate that IL-17 can participate in neoangiogenesis [89]. Nonetheless, it is important to underline that, while it can directly act as an angiogenic factor, IL-17 in combination with IFN*γ* increases the secretion of potent antiangiogenic factors such as CXCL9 and CXCL10 by cancer cells. The levels of CXCL9 and CXCL10 were associated with tumor-infiltrating effector T cells and improved outcomes in patients with ovarian cancer [37].

#### 3.1.3. IL-21, IL-22, TNF*α*, and IFN*γ*

As outlined, the cytokine secretion profile of Th17 cells is variable in nature and amount. We will therefore focus on the key factors produced by Th17 lymphocytes, which may influence anti-tumor immunity.

As mentioned in the previous section, IL-21 is involved in the generation of Th17 lymphocytes and is also produced by these cells. IL-6-induces IL-21 production in a STAT3-dependent and ROR*γ*-independent manner. IL-17 and IL-21 production is impaired *in vivo* in IL-6-deficient mice [48]. IL-21 can synergize with IL-12 to enhance the cytotoxicity of peripheral blood mononuclear cells (PBMC) in patients with cervical intraepithelial neoplasia III and cervical cancer. In this study, the PBMC incubated with IL-21 and IL-12 effectively induced apoptosis of SiHa tumor cells [97]. Additionally a report by Søndergaard et al. demonstrated that the administration of IL-21 significantly hindered the growth of established subcutaneous B16 melanomas or Renca renal cell carcinomas. The anti-tumoral effect of IL-21 was mediated in this case by CD8^+^ T lymphocytes [98].

IL-22 belongs to the IL-10 family and has often been reported as a cytokine produced by Th17 lymphocytes [99, 100]. In humans, IL-22 was initially characterized as a Th1 cytokine [101]. It was also reported that IL-22 could be secreted by CD4^+^ T cells in the absence of IL-17 production [102]. The possibility of the existence of a dedicated IL-22 secreting CD4^+^ T cell lineage (Th22) has been raised and whether “Th22” may belong to the Th17 family is currently being discussed [102–105]. Actually, it appears that naïve CD4^+^ T lymphocytes in the presence of IL-6 but in the absence of exogenous TGF*β* express high levels of IL-22 but minimal amount of IL-17 while IL-6 in combination with TGF*β* triggers the polarization of “conventional” Th17 lymphocytes expressing large amounts of IL-17 but minimal levels of IL-22 [106]. These IL-22 secreting cells have been described for their protective function against infections [107]. However, the presence of IL-22-producing CD4^+^ T cells has been correlated with poor survival in patients with gastric cancer [108]. IL-22 by itself has been described for both its pro- and anti-tumoral effects [103, 104, 109–111].

GM-CSF (granulocyte macrophage-colony stimulating factor) is endowed with anti-tumoral properties [112, 113]. It has been documented that GM-CSF is produced by highly pathogenic and proinflammatory Th17 cells in the setting of autoimmune diseases [114, 115]. GM-CSF production was dependent on the activity of the IL-12-IL-23 receptor complex and ROR*γ*t. Conversely, IFN*γ*, IL-12, and IL-27, known to inhibit ROR*γ*t expression, impeded GM-CSF secretion [114].

Th17 or hybrid Th17/Th1 lymphocytes can produce TNF*α* and IFN*γ* [38, 57]. Human tumor-infiltrating Th17 cells have been reported to produce high levels of TNF*α* and IFN*γ* [37]. These two cytokines are endowed with direct cytotoxic or cytostatic effects against tumor cells but are also involved in the activation of innate and adaptive immune cells, thus promoting anticancer immunity. Although TNF*α* is not essential for Th17 generation, it synergizes with IL-6 and IL-1*β* to amplify Th17 responses [67]. A significant positive correlation between the expression of genes involved in the TNF*α* signaling and those involved in Th17 pathways in patients with ovarian cancer was reported [42]. IFN*γ* is the hallmark of Th1 lymphocytes while Th17 cells generated *in vitro* typically produce minimal amounts of IFN*γ*. However, Th17 cells generated *in vivo*, especially during the development of autoimmune diseases, or adoptively transferred IL-17^+^ Th17 cells can evolve towards IL-17^+^ IFN*γ* ^+^ cells [36, 40, 116].

It should, however, be emphasized that conclusions drawn from the studies focusing on the effects of IL-17 should not be confused with those of Th17 cells since, as outlined above, several other non-CD4^+^ T cell populations can produce this cytokine. Similar considerations hold true for other Th17-related cytokines such as IL-21, IL-22, GM-CSF, TNF*α*, or IFN*γ*.

### 3.2. Th17 Lymphocytes in Cancer: Foes or Allies?

**Figure 2:**
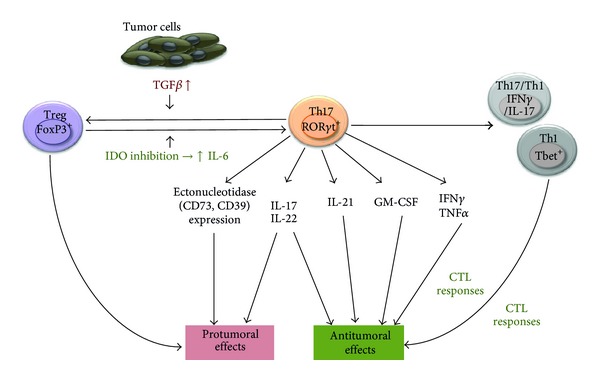
Pro-versus anti-tumoral effects of Th17 lymphocytes and the cytokines they produce on cancer development. Th17 lymphocytes produce cytokines which may promote or impair tumor development. Depending on the microenvironment Th17 may differentiate into Th1 or hybrid lymphocytes capable of controlling tumor growth or into protumoral Treg. IDO: indoleamine 2,3-dioxygenase.

As previously outlined, the role of Th17 lymphocytes in cancer is still highly controversial (Figure 2). An important distinction should be made between “endogenous” Th17 cells present in cancer patients or mouse tumor models, which develop under the pressure of the complex tumor environment, and the adoptively transferred Th17 cells generated *in vitro* under well-defined cytokine conditions.

Th17 lymphocytes have been detected in patients with different types of malignancies, such as ovarian, pancreatic, or gastric cancers, but the role of these cells in disease progression and their prognosis value has been controversial [37, 117, 118]. Whether Th17 lymphocytes are induced *de novo* from naïve CD4^+^ T cells or recruited at the tumor site or originate from “reprogrammed Treg” (see previous section and below) remains to be elucidated. In a report evaluating the nature of tumor-associated Th17 lymphocytes in ovarian cancer patients, it was demonstrated that the percentage of these cells correlated with the number of IFN*γ* ^+^ CD4^+^ T cells, IL-17^+^ IFN*γ* ^+^, IFN*γ* ^+^ CD8^+^ T cells as well as NK cells and inversely correlated with the frequency of immunosuppressive Treg cells [37]. In another report focusing on prostate cancer patients, highly differentiated Th17 cells correlated with slower disease progression [119], which was contradicted by results from others obtained in hormone resistant prostate cancer patients [120]. In additional studies, an association between increased numbers of tumor-associated Th17 lymphocytes and survival was observed in ovarian and lung cancer patients [37, 121]. Similarly, a significant increase in Th17 cell numbers in the tumor environment has been reported in the mouse ID8 ovarian, Pan02 pancreatic, and B16 melanoma cancer models [42, 122, 123]. The physiological significance of this increase has been disputed. In an ovarian cancer model, TNF*α*-mediated induction of IL-17-producing CD4^+^ cells led to the recruitment of myeloid cells into the tumor microenvironment and resulted in enhanced tumor growth [42]. In contrast, induced production of IL-6 in the tumor microenvironment, as a result of either indoleamine 2,3-dioxygenase (IDO) inhibition or the transduction of tumor cells with the IL-6 gene, led to the conversion of Treg to Th17 cells and regression of mouse B16 melanoma [123] or Pan02 pancreatic tumors [122], respectively.

Multiple studies have investigated the impact of *in vitro* generated Th17 cells on tumor growth following adoptive transfer, with variable outcomes. Initial studies by Muranski et al. have evaluated the effects of Th17 generated from CD4^+^ T cells isolated from TCR transgenic mice specific for the TRP-1 melanoma epitope. Administration of these Th17 lymphocytes led to the eradication of established B16 melanoma. The therapeutic effects of these cells were, however, substantially mediated by IFN*γ* [124]. In line with these results, subsequent studies indicated that adoptive transfer of *in vitro* generated Th17 lymphocytes impaired tumor development by eliciting robust tumor-specific CD8^+^ T cell responses. Th17 cell therapy promoted the homing of dendritic cells to the tumor site and the draining lymph nodes [41]. Supporting the anti-tumoral role of *in vitro* polarized Th17, in a more recent study Muranski et al. demonstrated that adoptive Th17 cell therapy has the potential to eliminate established tumors. The anti-tumoral efficacy of these Th17 lymphocytes was dependent on their ability to produce both IFN*γ* and IL-17. Interestingly, the administered Th17 differentiated into cells, which exhibited a stem cell-like phenotype and Th1 properties (Tbet, IFN*γ* expression) but retained their ability to produce IL-17. Importantly, the therapeutic efficacy of Th17 lymphocytes generated from Tbet^−/−^ or IFN*γ* ^−/−^ or IL17^−/−^ mice was severely impaired [74]. In agreement with these reports, our own results have suggested that the adoptive transfer of Th17 efficiently combined with chemotherapy to treat established murine mammary carcinoma [125]. In one study, the possibility that* in vitro* generated Th17 cells may exhibit immunosuppressive function and promote tumor progression through the expression of ectonucleotidases has been proposed [126].

It should, however, be emphasized that in the majority of these studies, the generated populations of CD4^+^ T cells were heterogeneous in nature and were not a pure subset of Th17 lymphocytes, advocating for a cautious interpretation of the above results. In addition, the concentration of cytokines (TGF*β*, IL-6, and IL-23) represents a major source of variability between protocols used to generate Th17 cells *in vitro.* Therefore, standardized procedures are still needed to generate and purify homogeneous populations of CD4^+^ T cells producing high levels of IL-17.

## 4. Conclusion: Manipulating the Differentiation Status of Th17 for Cancer Therapy

Although the role of Th17 in autoimmune diseases and infection has been relatively well documented, the impact of Th17 in cancer remains difficult to ascertain. The plasticity of the developmental program of these cells confers them with the ability to redifferentiate into suppressive Treg hindering anti-tumor immunity or alternatively into proinflammatory T helper cells such as Th1-like lymphocytes capable of activating tumor killer effector immune cells. This lack of clear lineage commitment explains the propensity of Th17 cells to be influenced in many different ways by the complex tumor microenvironment. The direction of Th17 eventual polarization is likely dictated by the concentration and ratio of cytokines and chemokines present in the tumor milieu, and by the presence and influence of other tumor-infiltrating immune cells. Since the tumor environment depends on the type, location, and stage of cancer, it is to be expected that Th17 function may vary according to these conditions. Controlling the level and type of the cytokines produced by cancer cells in animal tumor models may help addressing the conditions required for the pro- or anti-tumoral activity of Th17 lymphocytes. In addition, it would be essential to further evaluate the contribution of Th17 cells in tumor immunity at different stages of cancer progression. The degree of plasticity of these cells and their unpredictable behavior *in vivo* makes the prospect of Th17-based cancer immunotherapy highly challenging. However, based on the promising results obtained in preclinical animal models, the prospect of treating patients with Th-17 cells polarized *in vitro* seems an attractive strategy which deserves to be evaluated in clinical trials. The recent discovery of the stem cell-like properties of Th17, which enables them to self-renew with the capacity to differentiate into Th1-like or Treg progeny, could have significant implications on the outcome of Th17-based therapy. However, although IFN*γ*-expressing Th17 lymphocytes mediate potent anti-tumor effects both in human and animals, it will conceivably be challenging to consistently and reproducibly redirect Th17 differentiation towards IFN*γ*-expressing Th1-like cells following adoptive transfer* in vivo*. Further studies are therefore required to more clearly understand the driving forces sustaining Th17 polarization into potent anti-tumor effector cells.
